# The Msn2 Transcription Factor Regulates Acaricidal Virulence in the Fungal Pathogen *Beauveria bassiana*

**DOI:** 10.3389/fcimb.2021.690731

**Published:** 2021-07-20

**Authors:** Elen R. Muniz, Cárita S. Ribeiro-Silva, Walquíria Arruda, Nemat O. Keyhani, Éverton K. K. Fernandes

**Affiliations:** ^1^ Escola de Veterinária e Zootecnia, Universidade Federal de Goiás, Goiânia, Brazil; ^2^ Instituto de Patologia Tropical e Saúde Pública, Universidade Federal de Goiás, Goiânia, Brazil; ^3^ Instituto de Ciências Biológicas, Universidade Federal de Goiás, Goiânia, Brazil; ^4^ Department of Microbiology and Cell Science, University of Florida, Gainesville, FL, United States

**Keywords:** tick, biological control, entomopathogenic fungi, cuticle, virulence, *Beauveria bassiana*, *Rhipicephalus microplus*, Msn2

## Abstract

*Beauveria bassiana* holds promise as a feasible biological control agent for tick control. The *B. bassiana* stress–response transcription factor Msn2 is known to contribute to fungal growth, conidiogenesis, stress–response and virulence towards insects; however, little is known concerning whether Msn2 is involved in infection across Arthropoda classes. We evaluated the effects of Msn2 on *B. bassiana* virulence against *Rhipicephalus microplus* (Acari, Ixodidae) using wild-type, targeted gene knockout (*ΔBbmsn2*) and complemented mutant (*ΔBbmsn2/Bbmsn2*) strains. Reproductive parameters of *R. microplus* engorged females treated topically or by an intra-hemocoel injection of conidial suspensions were assessed. Treated cuticles of engorged females were analyzed by microscopy, and proteolytic activity of *B. bassiana* on cuticles was assessed. Topically treated engorged females showed high mean larval hatching (>84%) in control and *ΔBbmsn2* treatments, whereas treatment with the wild-type or *ΔBbmsn2/Bbmsn2* strains resulted in significantly decreased (lowered egg viability) larval hatching. Percent control of *R. microplus* topically treated with *ΔBbmsn2* was lower than in the groups treated with wild-type (56.1%) or *ΔBbmsn2/Bbmsn2* strains. However, no differences on reproductive parameters were detected when *R. microplus* were treated by intra-hemocoel injection using low (800 conidia/tick) doses for all strains tested; *R. microplus* injected with high doses of wild-type or mutant strains (10^6^ conidia/tick) died before laying eggs (~48 h after treatment). SEM analyses of *B. bassiana* infection showed similar conidial germination and formation of pseudo-appressoria on tick cuticle. Histological sections of ticks treated with the wild-type or *ΔBbmsn2/Bbmsn2* strains showed fungal penetration through the cuticle, and into the tick interior. Hyphae of *ΔBbmsn2*, however, did not appear to penetrate or breach the tick exocuticle 120 h after treatment. Protease activity was lower on tick cuticles treated with *ΔBbmsn2* than those treated with the wild-type or *ΔBbmsn2/Bbmsn2* strains. These data show that loss of the Msn2 transcription factor reduced *B. bassiana* virulence against *R. microplus*, but did not interfere with conidial germination, appressoria formation or sporulation on tick cadavers, and plays only a minimal role once the cuticle is breached. Our results indicate that the BbMsn2 transcription factor acts mainly during the fungal penetration process and that decreased protease production may be one mechanism that contributes to the inability of the mutant strain to breach the tick cuticle.

## Introduction

*Beauveria bassiana* (Hypocreales: Cordycipitaceae) is one of the most widely studied entomopathogenic fungi for applied tick control (Kirkland et al., 2004a; Kirkland et al., 2004b; Fernandes et al., 2012). The potential of this fungus to control *Rhipicephalus microplus* (Acari: Ixodidae) has been shown in laboratory assays, with variable virulence among *B. bassiana* isolates (Posadas and Lecuona, 2009; Campos et al., 2010; Fernandes et al., 2011; Sun et al., 2013). According to Fernandes et al. (2011), the mean lethal concentration of *B. bassiana* to kill 50% (LC_50_) of *R. microplus* engorged female can vary from 10^7^ to 10^9^ conidia ml^−1^.

*B. bassiana* conidia infect ticks through their cuticle or natural openings (Bernardo et al., 2018). The process of fungal infection on ticks is thought to be similar to that known for insects (Arruda et al., 2005), and includes conidial adhesion on the host cuticle, production of germ tube, differentiation into appressorium (something not seen for all *B. bassiana* isolates), penetration through the host cuticle by enzymatic action (e.g., lipases, proteases and chitinases) and mechanical pressure, and growth within the host integument and hemocoel (colonization stage) (Ortiz-Urquiza and Keyhani, 2016). The host dies by tissue destruction and by action of toxins from fungi (Schrank and Vainstein, 2010). However, ticks are known to potentially display significantly higher natural resistances to insect pathogenic fungi, and acaricidal specific factors may be produced by these fungi (Kirkland et al., 2004a, Kirkland et al., 2004b, Kirkland et al., 2005).

As mentioned, the virulence of *B. bassiana* is influenced by the production of proteases, particularly the subtilisin-like protease called Pr1 (Joshi et al., 1995), chitinases and lipases (Fang et al., 2005) and, specifically against ticks, the metabolite oxalic acid has been shown to be important (Kirkland et al., 2005). In the last fifteen years, many studies have contributed to understanding the network of genes which are related to the virulence of *B. bassiana* (Jin et al., 2010; Ortiz-Urquiza and Keyhani, 2013; Wang et al., 2013; Valero-Jiménez et al., 2016). Fang et al. (2005) demonstrated that overproduction of the *Bbchit1* chitinase enhanced the virulence of *B. bassiana* against aphids, as indicated by the significantly lower LC_50_ and mean lethal time to kill 50% (LT_50_) of target insects of the mutant strain compared to the wild-type strain. Another study using hybrid chitinase gene (Bb*chit1*–Bm*ChBD*) showed a 23% reduction of the LT_50_ to kill aphids (*Myzus persicae*) when they were treated with the mutant strain of *B. bassiana* in comparison to its wild-type (Fan et al., 2007).

Transcriptional regulation of effector genes in eukaryotic cells is one of the fundamental mechanisms involved in cellular responses to stress and/or virulence signaling pathways (Ortiz-Urquiza and Keyhani, 2015). In entomopathogenic fungi, the transcription factor Msn2 regulates the conidiogenesis of *B. bassiana* and *Metarhizium robertsii*; a knockout strain of each species (*ΔBbmsn2* and *ΔMrmsn2*, respectively) reduced 43 and 39% the conidial production in comparison to their respective wild-type strains (Liu et al., 2013). Also, *ΔBbmsn2* and *ΔMrmsn2* strains had a reduced cell tolerance to chemical and environmental stresses. Decreased virulence of *ΔBbmsn2* and *ΔMrmsn2* against *Spodoptera litura* (Lepidoptera: Noctuidae) second-instar larvae and *Tenebrio molitor* (Coleoptera: Tenebrionidae) third-instar larvae was recorded; the LT_50_ values were 28 and 25% longer than the control strains of *B. bassiana* (5.5 days) and *M. robertsii* (4.8 days), respectively. In *Galleria mellonella* (Lepidoptera: Pyralidae) larvae, the LT_50_ significantly increased when treated with the knockout strain (LT_50_ = 3.4 ± 0.08 days) in comparison to the wild-type strain (2.71 ± 0.03 days) (Luo et al., 2015). These data confirmed that Msn2 is critical for entomopathogenic fungal infection of insects; however, any similar role towards Acari has not been investigated. Furthermore, to date, no histological investigations related to *Msn2* on infection or effects on reproduction have been performed on insects or ticks. Overall, little is known about the molecular basis of virulence in *B. bassiana* towards ticks. Here, we examined the consequences of loss of *Msn2* on *B. bassiana* infection towards ticks combining genetic characterization with enzymatic and histological approaches. Our data indicate *BbMsn2* is critical for penetration but not subsequent growth and proliferation once the tick cuticle has been breached. Furthermore, important effects were seen with respect to lowered female fertility, indicating potential added benefits in the application of *B. bassiana* for tick control.

## Material and Methods

### *Beauveria bassiana* Strains, Conidial Suspensions, and Viability

The strains of *B. bassiana* knockout *ΔBbmsn2* and complemented (*ΔBbmsn2*/*Bbmsn2*) were constructed and initially characterized by Luo et al. (2015). *ΔBbmsn2* and the complemented strain were obtained from *B. bassiana* Bb0062. *Beauveria bassiana* strains were cultivated on potato dextrose agar (PDA, Difco Laboratories, Sparks, MD, USA) supplemented with 1 g L^−1^ yeast extract (Bacto™ Yeast Extract, Sparks, MD, USA) (PDAY) in Petri plates (90 × 15 mm) and incubated in the dark for 15 days at 26 ± 1°C and relative humidity (RH) ≥90%. Temperature and relative humidity in the incubator were monitored with a data logger HOBO H8^®^ (Onset Computer Corporation, Bourne, MA, USA). Fresh conidia from each strain were harvested using a spatula, suspended in 0.01% (v/v) Tween 80^®^ (Labsynth Prod. Lab. Ltda, Diadema, SP, Brazil) and filtered through cheesecloth to remove mycelia. Conidial suspensions were quantified in hemocytometer at 400× magnification in a Leica DM750 light microscope (Leica Microsystems, Wetzlar, Germany), and the concentration was adjusted to 2.0 × 10^8^ conidia ml^−1^ or as indicated. To assess conidial viability, 20 µl of each conidial suspension were inoculated onto 8 ml of PDAY medium supplemented with 0.002% (w/v) Benomyl (50% active ingredient; Benlate^®^, DuPont, São Paulo, SP, Brazil) (Braga et al., 2001) and 0.05% (w/v) chloramphenicol (Sigma-Aldrich, Steinheim, Germany) in Petri plates (35 × 9 ×10 mm). The plates were incubated at 26 ± 1°C for 48 h. After incubation, a drop of lactophenol and cotton blue solution and coverslip were applied over the inoculum; a minimum of 300 conidia per plate was evaluated, and the percent relative germination was determined (Braga et al., 2001). Conidia were considered germinated when the germ tube was longer than the maximum conidial diameter. Conidia were used only if viability was assessed to be >98% in all experiments.

### Fungal Bioassays Using *Rhipicephalus microplus* and Measurement of Tick Reproductive Parameters

*Rhipicephalus microplus* engorged females were collected from artificially infested cattle at Universidade Federal de Goiás (UFG, Goiânia, Brazil). In the laboratory, ticks were washed in tap water, immersed in 1% (v/v) hypochlorite for 1 min, rinsed in sterile distilled water for 1 min and dried with sterile paper towels. The females were homogeneously distributed by weight (160-315 mg) into four treatment groups: control, *BbWT*, *ΔBbmsn2/Bbmsn2* and *ΔBbmsn2*; each group had 10 individuals.

Two infection protocols were evaluated: (i) topical and (ii) intra-hemocoel injection. For topical assays, ticks were individually immersed in 1 ml of Tween 80^®^ 0.01% (control) or in the conidial suspensions at 2.0 × 10^8^ conidia ml^−1^ for 3 min. In addition, ticks were individually inoculated by intra-hemocoel injection with 5 µl of Tween 80^®^ 0.01% (control), or 5 µl of conidial suspension at 1.6 × 10^5^ conidia ml^−1^ (800 conidia/tick), 1.6 × 10^6^ conidia ml^−1^ (8,000 conidia/tick), 1.6 × 10^7^ conidia ml^−1^ (80,000 conidia/tick) or 2.0 × 10^8^ conidia ml^−1^ (10^6^ conidia/tick); injections were performed in the foramen located between the capitulum and the dorsal scutum of engorged females using a stereomicroscope and a 0.3 mm insulin syringe (Angelo et al., 2010). After treatment, ticks were individually placed in each well of 24-well cell culture plates (Corning Brasil Indústria e Comércio Ltda., Suzano, SP, Brazil), incubated at 26 ± 1°C, RH ≥90% and 12 h photophase. The egg mass from each female was collected at the end of oviposition, weighed, transferred to an individual glass tube (16 × 125 mm), closed with a cotton plug, and incubated at 26 ± 1°C and RH ≥90% for assessment of larval hatching. The eggs were observed daily and the larval hatching for each tube was visually estimated through microscopic examination, and values were assigned in percentages ranging from 0 to 100% by intervals of 5% in relation to the total mass of eggs (Drummond et al., 1971; Barreto et al., 2016; Bernardo et al., 2018).

The following reproductive parameters were investigated: estimated reproduction (ER) and percent control (PC) (Drummond et al., 1971). The effectiveness of treatment was measured by the effect on the ER of the engorged females; in this equation, 20,000 is the estimate of the number of larvae that normally hatch from 1 g of eggs of *R. microplus*; therefore, the percent control of ER estimates the treatment efficacy to decrease the tick population in an infested environment. Bioassays were repeated three times on different days, and with new batches of conidia. The ER and PC were calculated by the Equations (1) and (2), respectively.

Equation (1):

$$ER=\frac{weight\;of\;egg\;mass\;(g)}{initial\;weight\;of\;engorged\;female\;(g)}\times percentage\;of\;larval\;hatch\;\times 20,000$$

Equation (2):

$$PC=\frac{mean\;ER\;of\;the\;control\;group\;-mean\;ER\;of\;the\;treated\;group}{mean\;ER\;of\;control\;group}\times 100$$

### Scanning Electron and Light Microscopy of Tick’s Cuticle

Initial steps of the *B. bassiana* infection process were examined by scanning electron microscopy (SEM) and light microscopy. Six *R. microplus* engorged females were individually treated with *BbWT*, *ΔBbmsn2/Bbmsn2* or *ΔBbmsn2* by topical application of 2.5 µl conidial suspension at 2.0 × 10^8^ conidia ml^−1^. The treated females were incubated at 26 ± 1°C, with RH ≥90% and 12 h photophase, for 48 or 120 h. Then, approximately 50 µl of fixative [2% (v/v) glutaraldehyde (Impex, Labimpex Ind. Com. de Prods. Lab. Ltda., Diadema, SP, Brazil), 2% (v/v) paraformaldehyde (Vetec Química Fina Ltda, Duque de Caxias, RJ, Brazil), 3% (w/v) sucrose (Sigma-Aldrich, Steinheim, Germany) in 0.1 M sodium cacodylate buffer (Sigma-Aldrich, Steinheim, Germany), pH 7.2] was injected into each female using an insulin syringe according to Barreto et al. (2016). Each female was placed in a 15-ml centrifuge tube containing 2 ml of fixative and maintained for 10 days at 4°C in a refrigerator.

Ticks incubated for 48 h were examined by SEM (n = 3). The dorsal cuticle of females was dissected and removed, and then washed three times (15 min each time) in sodium cacodylate buffer (0.1 M, pH 7.2). Cuticles were dehydrated in a graded series of ethanol solutions (30, 50, 70, 80 and 90%), held for 15 min in each solution and passed twice in 100% ethanol for 15 min. Subsequently, the cuticles were individually placed in micro-centrifuge tubes containing 300 µl of hexamethyldisilazane (Electron Microscopy Sciences, Hatfield, PA, USA) and maintained immersed for 5 min (Barreto et al., 2016). After drying, the samples proceeded to metallization. Accordingly, the samples were placed on a stub and coated with gold in a sputter-applicator (Denton Vacuum Desk V). The cuticles were analyzed and electro micrographs were obtained with a scanning electron microscope (Jeol JSM 6610) at an accelerating voltage of 20 kV. The images were analyzed qualitatively (conidial adhesion, size of germinative tubes and presence of appressoria) and quantitatively (number of germinated conidia).

Ticks incubated for 120 h were prepared for histological analyses of their cuticle. After 10 d in fixative, treated ticks (n = 3) were longitudinally cut, dehydrated as described with SEM samples and embedded in resin (Historesin^®^, Leica Biosystems, Wetzlar, Germany) according to the manufacturer’s instructions. Sections of 4 µm were made in Microtome (Leica Biosystems, Wetzlar, Germany), stained with Periodic Acid-Schiff (PAS) and Green light (Arruda et al., 2005) and assessed by using a light microscope (Nikon E200). Images (n = 5) were captured with a high-definition microscope camera Leica ICC50 HD, with resolution of 1,280 × 720 p (HD ready). The experiments were conducted three times on different days with three replicates in each treatment group.

### Protease Assay

Cuticles of *R. microplus* engorged females were dissected (n = 10), washed in distilled water and immersed in 10 ml conidial suspension of the strain *BbWT*, *ΔBbmsn2/Bbmsn2* or *ΔBbmsn2*, at 2.0 × 10^8^ conidia ml^−1^ for 3 min. Cuticles were incubated at 26°C and RH ≥90% for 120 h. After incubation, the pool of cuticles was macerated in 1 ml distilled water and centrifuged at 25,000 RCF for 5 min at 4°C. Protease activity was measured by azocasein hydrolysis as described by Segers et al. (1994) and Phillips et al. (1984) with the following modifications: commercial azocasein (Sigma Chemical Co., St. Louis, MO, USA) was dissolved at 1% (w/v) in 0.1 M Tris–HCl buffer, pH 8.5. Briefly, 1,000 µl of each supernatant sample were incubated with 500 µl de azocasein 1% at 28°C for 60 min. This reaction was stopped by adding 1 ml of 10% TCA and maintaining it for 15 min at 4°C, and followed by centrifugation at 25,000 RCF for 10 min to remove the precipitated protein. Six hundred milliliters of the supernatant were neutralized by adding 700 µl of 1M NaOH, and absorbance at 450 nm was recorded with Enzyme-Linked Immunosorbent Assay (ELISA). One unit of enzyme activity (U) was calculated by the Equation (3):

Equation (3):

$$\text{UA}=\frac{\text{control}\;\text{absorbance}\;-\;\text{sample}\;\text{absorbance}}{0.001}\times \frac{1}{60}$$

### Statistical Analyses

All data sets were previously checked for normality and homoscedasticity with Shapiro–Wilk and Bartlett tests, respectively. Normally distributed data (engorged female initial weight, egg mass weight, and conidial germination) were fitted to a parametric model and then were submitted to ANOVA followed by an SNK test for multiple comparisons. Non-normally distributed data (percentage of larval hatch) were fitted to a non-parametric model and then analyzed by a Kruskal–Wallis test, followed by an FDR test. Protease activity of mutant strains was compared with the *BbWT* strain by applying the Dunnett’s test. Analyses were performed in the statistical environment R (R Team C, 2018). *P*-values less than 0.05 were considered as significant.

## Results

### Measurement of Reproductive Parameters and Virulence Assays

Reproductive parameters of *R. microplus* engorged females treated topically with different strains (*BbWT*, *ΔBbmsn2*, and *ΔBbmsn2/Bbmsn2*) of *B. bassiana* were examined 15 d post-infection after immersion in 2.0 × 10^8^ conidia ml^−1^ as detailed in the *Material and Methods* section (**Table 1**). Because of the difficulty in determining the exact timing of tick death, lethal mortality times could not be accurately determined; however, reproductive parameters that would critically inform on successful biological control efforts were measured. The weight of egg mass from females treated with any of the *B. bassiana* conidial suspensions tested (i.e., either wild-type, mutant, or complemented mutant) was similar and lower (~50%) than that of control untreated ticks (*F*_3,118_ = 18.33; *P <*0.001). High mean larval hatching (~90%) was observed in the control group, which was slightly reduced after treatment with the *ΔBbmsn2* strain (down to ~84%, *P* = 0.006). However, engorged females treated with *BbWT* or *ΔBbmsn2/Bbmsn2* had significantly decreased larval hatching (10–20%, χ^2^ = 17.38; *df* = 3; *P* = 0.006), indicating lower egg viability using the wild-type and complemented *B. bassiana* strains. Overall, the percent control of ticks was higher in the groups treated with *BbWT* (56.1%) or *ΔBbmsn2/Bbmsn2* (58.7%) as compared to untreated engorged females or ticks treated with the *ΔBbmsn2* mutant strain (39.7%). Despite being unable to determine exact time of death, all ticks showed eventual mycosis and sporulation of the fungus on the tick cadavers.

**Table 1:** Biological parameters of *Rhipicephalus microplus* engorged females treated by immersion in Tween 80^®^ 0.01% (control) or in conidial suspension (2.0 × 10^8^ conidia ml^−1^) of *Beauveria bassiana* strains (*BbWT*, *ΔBbmsn2/Bbmsn2* or *ΔBbmsn2*) incubated at 26 ± 1°C and RH ≥ 90%.

Treatment by immersion	Engorged female weight (mg)	Egg mass weight (mg)	Larval hatch (%)	ER^†^	Percent control (%)^‡^ (n = 30)
Control	232.1 ± 16^a^	126.3 ± 15^a^	89.2 ± 3.2^a^	968,730.1	–
*BbWT*	231.2 ± 16^a^	66.4 ± 13^b^	78.5 ± 3.0^bc^	447,094.8	56.1
*ΔBbmsn2/Bbmsn2*	233.4 ± 14^a^	68.2 ± 15^b^	70.9 ± 4.9^c^	406,020.6	58.7
*ΔBbmsn2*	232.5 ± 16^a^	77.6 ± 16^b^	84.0 ± 2.2^ab^	554,807.5	39.7

(†) ER: estimated reproduction [ER = weight of egg mass (g)/initial weight of engorged female (g) × percentage of larval hatch × 20,000] (Drummond et al., 1971).

(‡) Percent control [ER of the control group − mean ER of the treated group/mean ER of the control group × 100] (Drummond et al., 1971). Means are followed by standard errors of 10 replicates per bioassay, in three independent trials. Means followed by the same letter in the same column did not differ significantly (P > 0.05) according to the Student–Newman–Keuls (engorged female weight and egg mass weight) or Kruskal–Wallis test (larval hatch).

**Figure 1:**
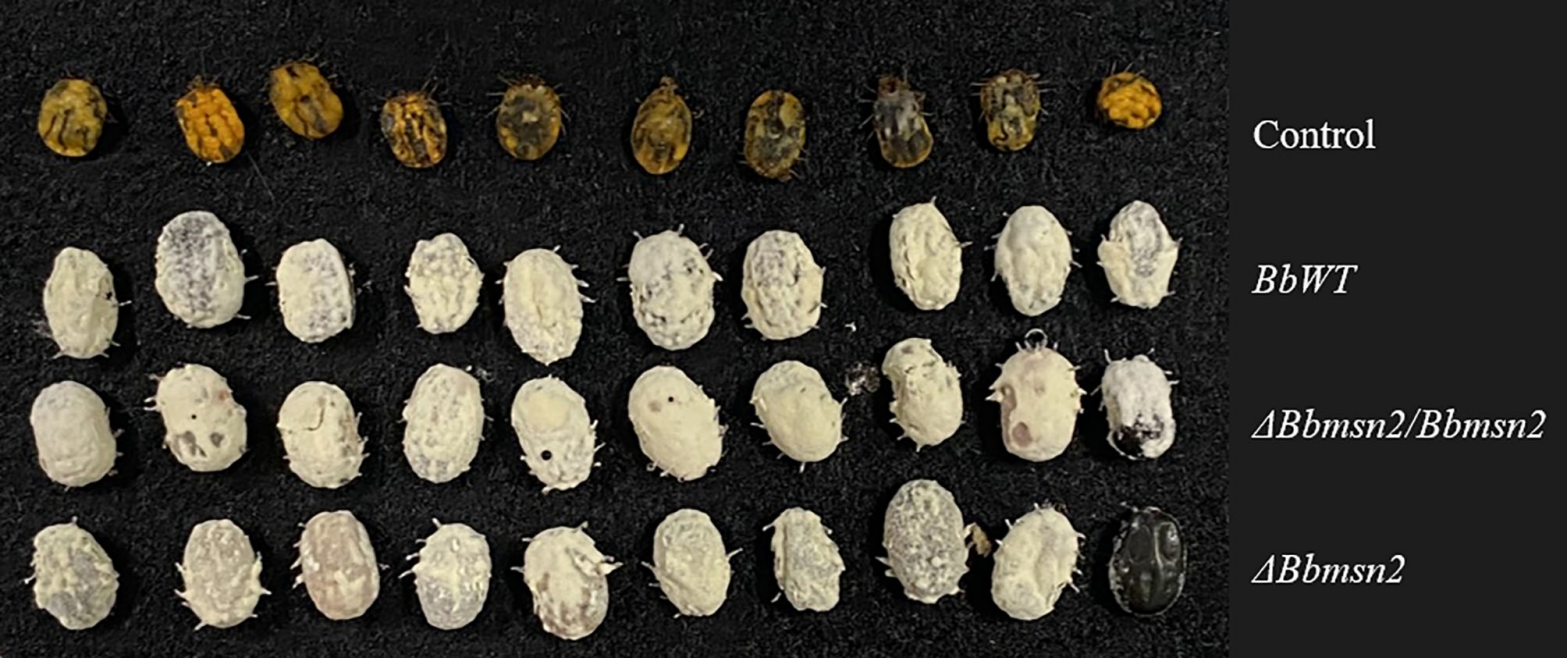
Fungal sporulation on *Rhipicephalus microplus* engorged females’ cadavers at day 5 post treatment by an intra-hemocoel injection with 5-μl conidial suspension at 2.0 × 10^8^ conidia ml^–1^.

For intra-hemocoel injection assays (that would bypass the need to cuticle penetrations), a concentration range of fungal conidia (800, 80,000, 80,000, and 10^6^ conidia/tick) was tested (**Table 2**). Engorged females of *R. microplus* treated by intra-hemocoel injection of 800 conidia/tick showed decreased (50–60%) mean egg mass weights for all strains tested (*F*_3,55_ = 24.05; *P <*0.001) as compared to control uninfected ticks, with only slight differences seen between the wild-type, *ΔBbmsn2*, and *ΔBbmsn2/Bbmsn2* strains (**Table 2**). In addition, a decreased mean oviposition period from 7 to 2 d was seen after injection of 800 conidia/tick of any of the *B. bassiana* strains tested (*F*_3,55_ = 27.66; *P <*0.001) in comparison to control untreated ticks. Ticks treated by intra-hemocoel injection at concentrations ≥8,000 conidia/tick died before laying their eggs (i.e., within 48 h after treatment), while in control group the oviposition period was 7.9 ± 1 d (**Table 2**), and thus had essentially no oviposition period. No differences in sporulation were observed on the cadaver of ticks between the wild-type, *ΔBbmsn2*, and *ΔBbmsn2/Bbmsn2* strains at 5 d after infection (**Figure 1**).

**Table 2:** Biological parameters of *Rhipicephalus microplus* engorged females treated by an intra-hemocoel injection with 5 µl of Tween 80^®^ 0.01% (control), or 5 µl of conidial suspension of *Beauveria bassiana* strains (*BbWT*, *ΔBbmsn2/Bbmsn2* and *ΔBbmsn2*) at 1.6 × 10^5^ conidia ml^−1^ (800 conidia/tick), 1.6 × 10^6^ conidia ml^−1^ (8,000 conidia/tick), 1.6 × 10^7^ conidia ml^−1^ (80,000 conidia/tick) or 2.0 × 10^8^ conidia ml^−1^ (10^6^ conidia/tick), and incubated at 26 ± 1°C and RH ≥ 90%.

Treatment by inoculation	Dosage (conidia/tick)	Engorged female weight (mg)	Egg mass weight (mg)	Oviposition period (days)*
Control	0	249.1 ± 0.6^a^	78.1 ± 19 (n = 30)	7.9 ± 1
* BbWT*	10^6^	249.0 ± 1.1^a^	0 (n = 0)	–
*ΔBbmsn2/Bbmsn2*	10^6^	250.4 ± 1.6^a^	0 (n = 0)	–
*ΔBbmsn2*	10^6^	251.6 ± 0.9^a^	0 (n = 0)	–
Control	0	236.1 ± 6.0^a^	76.4 ± 3.9 (n = 30)	7.9 ± 0.4
*BbWT*	8 × 10^4^	239.3 ± 6.6^a^	0 (n = 0)	–
*ΔBbmsn2/Bbmsn2*	8 × 10^4^	237.5 ± 5.7^a^	0 (n = 0)	–
*ΔBbmsn2*	8 × 10^4^	232.3 ± 6.4^a^	0 (n = 0)	–
Control	0	235.6 ± 6.8^a^	77.8 ± 5 (n = 30)	6.7 ± 0.1
* BbWT*	8 × 10^3^	239.2 ± 7.0^a^	0 (n = 0)	–
*ΔBbmsn2/Bbmsn2*	8 × 10^3^	236.4 ± 7.8^a^	0 (n = 0)	–
*ΔBbmsn2*	8 × 10^3^	237.1 ± 6.6^a^	0 (n = 0)	–
Control	0	237.9 ± 8.2^a^	76.8 ± 5a (n = 30)	7.2 ± 0.5^a^
*BbWT*	800	237.6 ± 9.1^a^	32.7 ± 6b (n = 7)	2.3 ± 0.1^b^
*ΔBbmsn2/Bbmsn2*	800	239.6 ± 9.0^a^	11.0 ± 1b (n = 11)	2.0 ± 0.0^b^
*ΔBbmsn2*	800	238.9 ± 9.0^a^	14.6 ± 5b (n = 12)	2.1 ± 0.05^b^

(*) Mean number of days engorged females laid their eggs. Means are followed by standard errors of 30 replicates per bioassay. Means followed by the same letter in the same column, and in the same dosage tested, did not differ significantly (P > 0.05) according to the Student–Newman–Keuls test.

### Infection of *B. bassiana* Strains on Tick Cuticle

**Figure 2:**
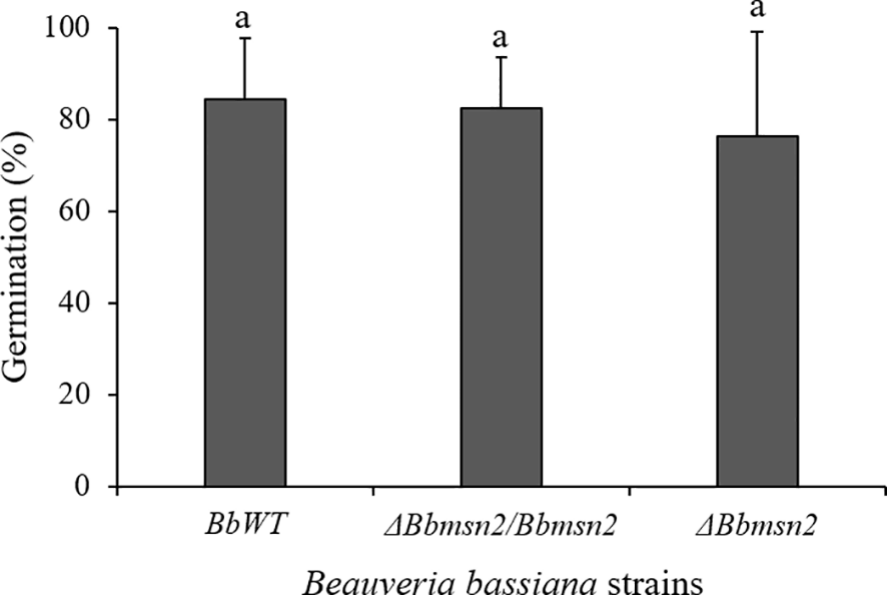
Germination (%) of conidia of *Beauveria bassiana* strains: *BbWT*, *ΔBbmsn2/Bbmsn2* and *ΔBbmsn2* on the cuticle of *Rhipicephalus microplus* engorged females incubated at 26 ± 1°C and RH ≥90% for 48 h. Bars with the same letter do not differ significantly (*P* ≥ 0.05) among treatments. Standard errors are based on three independent trials.

**Figure 3:**
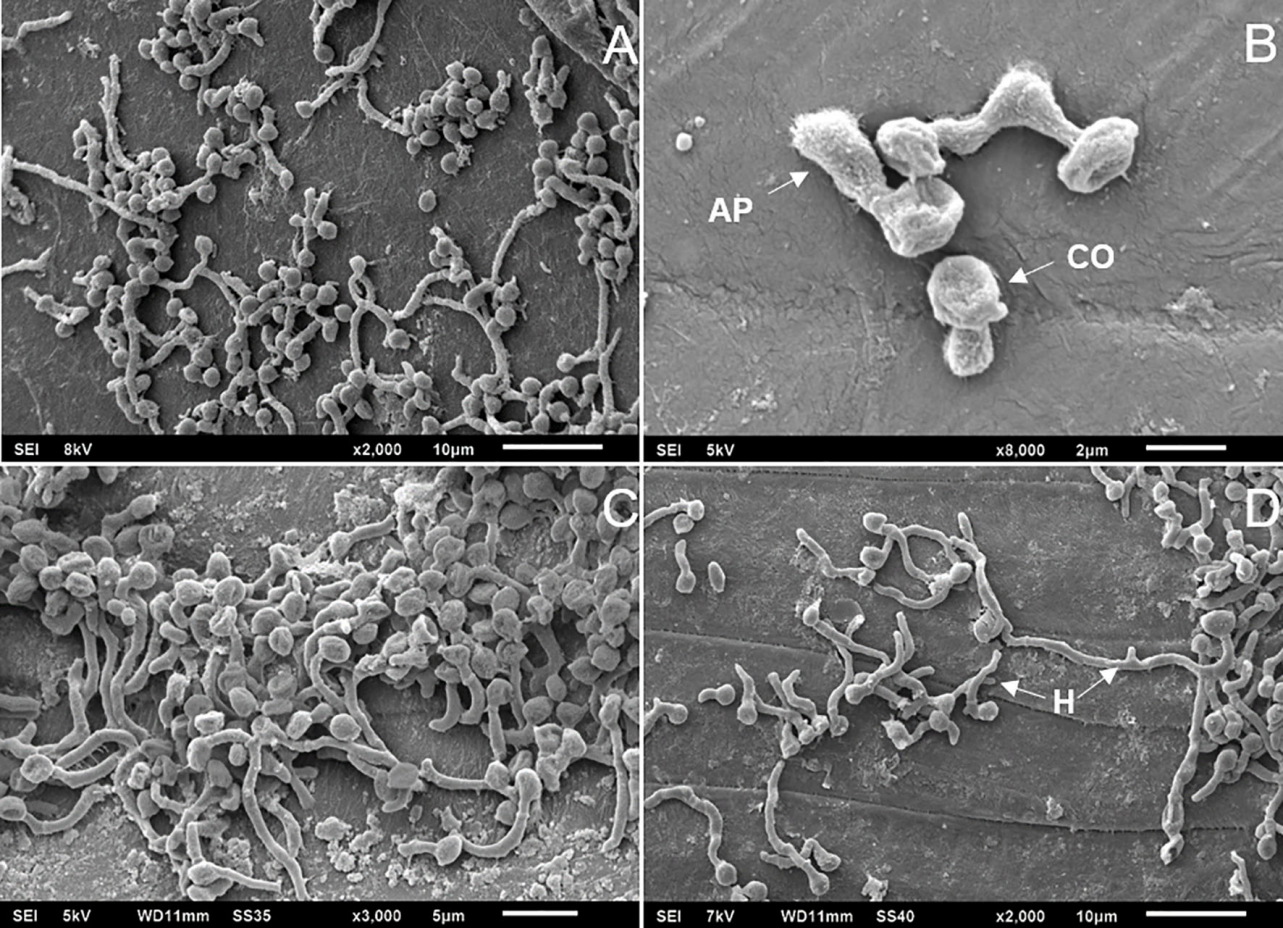
Electron micrographs of *Rhipicephalus microplus* engorged females treated topically with conidia of *Beauveria bassiana* strains: *BbWT*, *ΔBbmsn2/Bbmsn2* or *ΔBbmsn2*, and incubated for 48 h at 26 ± 1°C and RH ≥ 90%. Germinating conidia of *BbWT* on the tick cuticle **(A)** Germinating conidia of *ΔBbmsn2/Bbmsn2* with developed appressorium on the tick cuticle **(B)** Germinating conidia of *ΔBbmsn2/Bbmsn2* on the tick cuticle **(C)** Germinated conidia of *ΔBbmsn2* [some with long germ tubes (hyphae)] on the tick cuticle **(D)**. CO, conidia; AP, appressorium; H, hyphae.

The absence of the Msn2 transcription factor did not interfere with conidial germination or adhesion to the tick cuticle. Electron micrographs showed similar (*F*_2,6_ = 0.0638; *P* = 0.9388) conidial germination between the *B. bassiana* strains on the tick cuticle at 48 h incubation: *BbWT* = 84.33 ± 13.2%, *ΔBbmsn2/Bbmsn2* = 82.33 ± 11.20% and *ΔBbmsn2* = 76.33 ± 22.67% (**Figure 2**). Most conidia from *BbWT* (**Figure 3A**), *ΔBbmsn2/Bbmsn2* (**Figures 3B, C**) and *ΔBbmsn2* (**Figure 3D**) appeared to have germinated, and appressoria-like tip structures could be seen.

**Figure 4:**
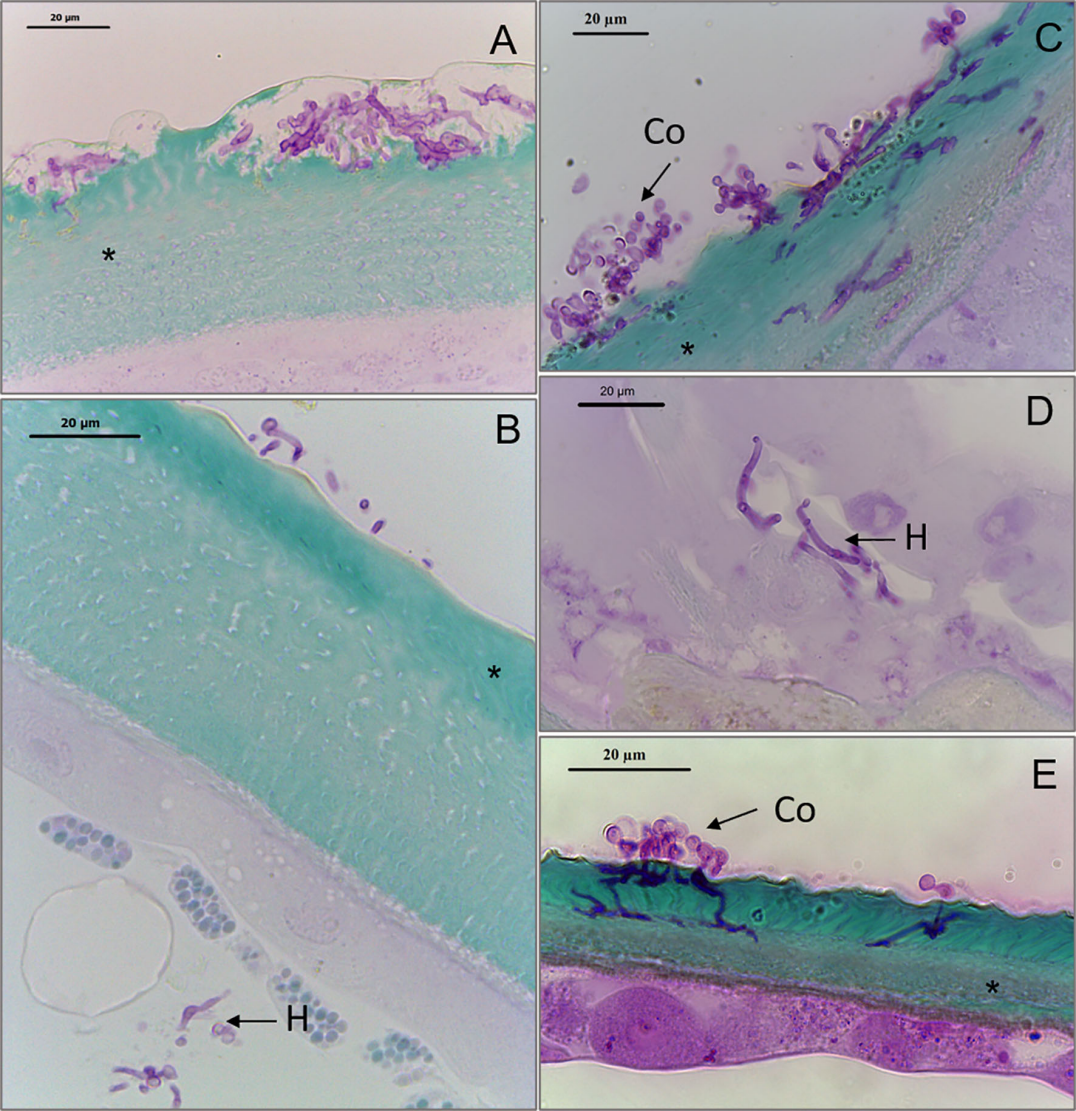
Sagittal sections of *Rhipicephalus microplus* engorged females treated with conidia of *Beauveria bassiana* strains: *BbWT*, *ΔBbmsn2/Bbmsn2* or *ΔBbmsn2*, and incubated for 120 h at 26 ± 1°C and RH ≥90%. Germinating conidia of *BbWT* penetrates through the tick cuticle **(A)** Germinating conidia of *BbWT* attaches to the tick cuticle and fungal hyphae infecting the tick interior **(B)** Germinating conidia of *ΔBbmsn2/Bbmsn2* penetrates through all the layers of the tick cuticle **(C)** Hyphae of *ΔBbmsn2/Bbmsn2* infects the interior tick tissues **(D)** Incomplete penetration of *ΔBbmsn2* hyphae through the tick cuticle **(E)**. Co, germinated conidia; H, hyphae inside tick tissue; asterisk (*) cuticle.

Conidia from *BbWT* and *ΔBbmsn2/Bbmsn2* were capable of germinating and penetrating through the cuticle (**Figures 4A, C**, respectively). Hyphae of *BbWT* (**Figures 4A, B**) and *ΔBbmsn2/Bbmsn2* were also observed inside the tick body, with fungal development in adjacent tissues (**Figures 4C, D**). However, the hyphae of the *ΔBbmsn2* strain appeared to continue to grow on the surface/exocuticle of the tick with fewer instances of penetrative hypha seen at 120 h incubation as compared to the wild-type and complemented strains (**Figure 4E**).

### Protease Activity

**Figure 5:**
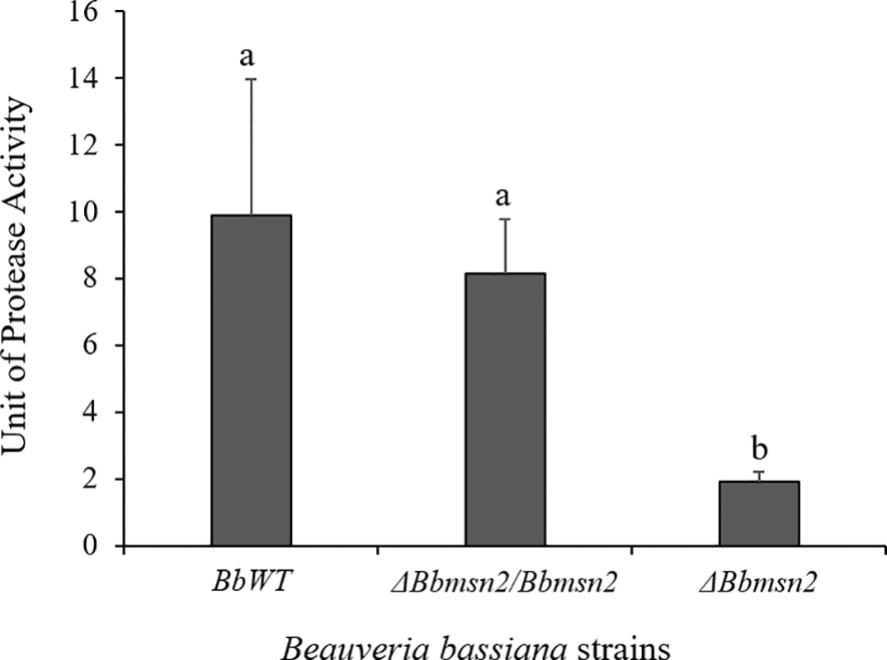
Protease activity on the cuticle of *Rhipicephalus microplus* treated by an immersion in a conidial suspension of *Beauveria bassiana* strains: *BbWT, ΔBbmsn2/Bbmsn2* or *ΔBbmsn2*, and incubated for 120 h at 26 ± 1°C and RH ≥90%. Bars with different letters, i.e. “a” and “b”, indicate significant difference (*P* < 0.05). Standard errors are based on three independent trials.

The proteolytic activity of the *B. bassiana* strains on *R. microplus* cuticle was measured using azoalbumin as detailed in the *Material and Methods* section. Protease activity was significantly lower in the *ΔBbmsn2* strain (1.91 ± 0.29 U ml^−1^) than in the *BbWT* (9.88 ± 4.08 U ml^−1^; *P* = 0.0113) and *ΔBbmsn2/Bbmsn2* strains (8.16 ± 1.59 U ml^−1^; *P* = 0.0150) (**Figure 5**).

## Discussion

Our data indicate that the Msn2 transcription factor significantly contributes to the ability of *B. bassiana* to infect *R. microplus via* the “natural” cuticle-penetration requiring route. Engorged females treated topically with the *ΔBbmsn2* strain showed a significantly decreased percent control of ticks in comparison to the wild-type and complemented strains. However, the mortality of ticks reached 100% when engorged females were injected, thus by-passing the requirement for cuticle penetration, with *ΔBbmsn2* (≥8,000 conidia/tick) conidial suspension by intra-hemocoel injection. Even at the lowest dose injected (800 conidia/tick), reduced reproductive parameters of engorged females was seen for all of the *B. bassiana* strains tested. In a previous study, *S. litura* and *T. molitor* larvae treated with *ΔBbmsn2* conidia had, respectively, LT_50_ values 28 and 25% higher (decreased virulence) than the complemented strain (Liu et al., 2013). In addition, *G. mellonella* larvae treated topically with *ΔBbmsn2* conidia also had an increased LT_50_ detected; however, no difference in LT_50_ values among the mutant and wild-type strains was observed when *G. mellonella* larvae were treated by injecting conidial suspension into the hemocoel (Luo et al., 2015). These results and our data lead us to conclude that the transcription factor Msn2 acts predominantly during the penetration of *B. bassiana* through the tick cuticle, with retention of the ability of the fungus to evade immune systems once inside the host.

The first step to successful infection of entomopathogenic fungi is the adhesion of fungal propagules (conidia or blastospores) on the host cuticle. Accordingly, high fungal virulence is directly related to increased adhesion to the host cuticle (Butt and Goettel, 2000). Adhesion is considered to follow a two-step process involving initial attachment followed by consolidation of adhesion, influenced by the surface characteristics of the fungal cells and the target substrata (Holder et al., 2007). The *B. bassiana* conidial surface is composed of a layer of hydrophobic rod-like proteins (hydrophobins, *hyd1* and *hyd2*), which mediate, in part, attachment to the host cuticle that is also hydrophobic (Holder and Keyhani, 2005; Ortiz-Urquiza and Keyhani, 2016). The deletion of *hyd1* gene in *B. bassiana* decreased the conidia hydrophobicity and virulence, although it did not interfere in its adhesion capacity. The deletion of the *hyd2* gene caused changes in the structure of the conidia cell wall, and consequently diminished its adhesion capacity and hydrophobicity but without altering its virulence (Zhang et al., 2011). Liu et al. (2013) have shown that the hydrophobin genes (*hyd1* and *hyd2*) were repressed by 66–68% in a *ΔBbmsn2* mutant strain, but it did not affect the *mad1* and *mad2* expressions, additional factors important for facilitating conidial adhesion to arthropods and plants, respectively (Broetto et al., 2010). Microscopic analyses were used in order to probe effects of BbMsn2 on adhesion to tick surfaces, and these data showed that conidia of *ΔBbmsn2* were equivalent to the wild-type (*BbWT*) and complemented strains (*ΔBbmsn2/Bbmsn2*) in size and ability to adhere to the tick cuticle. These data indicate that the impaired virulence seen in topical assays is not likely due to impaired adhesion to the host surface.

The tick cuticle is divided in several layers; from the outside to inside: epicuticle, exocuticle, endocuticle and epidermis (Hackman, 1982). The epicuticle layer is composed of lipids, long chain alkenes, esters and fatty acids (Ali et al., 2009). In particular, *R. microplus* females, during the engorgement process, have altered epicuticle composition that includes an almost 20% increase in surface lipids; with the most commonly found including alcohol and fatty acids combined by esterification (Hackman, 1982). To penetrate the host cuticle, entomopathogenic fungi use mechanical pressure and secrete proteases, chitinases, and lipases, which degrade their main constituents (proteins, chitin, and lipids) to allow the hyphae to penetrate through the exoskeleton. *B. bassiana* secretes lipases which potentiate the degradation of the arthropod wax layer (Sánchez-Pérez et al., 2014). However, Feng (1998) indicated that lipase activity of several *B. bassiana* s.l. isolates had little correlation with their virulence. Luo et al. (2015) demonstrated that proteinase and lipase activities were similar in *ΔBbmsn2* colonies grown on skim milk agar plates and in the wild-type. However, in the present study, our data show that the protease activity of *B. bassiana ΔBbmsn2* on *R. microplus* cuticle decreased in the Msn2 mutant. It is known that the production of (total) protease activity can vary significantly according to the composition of host cuticle (Cito et al., 2016), which may induce or inhibit fungal development (Ment et al., 2012). Additionally, Santi et al. (2019) identified 50 proteins involved in the infection process, which were produced by *B. bassiana* cultured in media supplemented with cuticles derived from *R. microplus*, but not produced in media supplemented with glucose only. Our results showed a decreased penetration of *ΔBbmsn2* hyphae through the cuticle in comparison to the wild-type and complemented strains at 120 h post-infection. This inability to trespass the tick cuticle may be due to the reduced capacity to degrade chitin, proteins, and/or lipids that constitute the cuticle composition of *R. microplus* (Hackman, 1982). In addition, impairments related to the fungal response to the osmotic, oxidative and/or nutrient stress that occurs on the cuticle, and are mediated by *Msn2*, may also contribute to the decreased virulence (Luo et al., 2015).

The production of oxalic acid is also an important virulence factor in *B. bassiana* against ticks. Direct treatment of ticks with oxalate at pH 4.0 resulted in almost 80% mortality in adults of the tick *Amblyomma americanum* within 14 days after treatment (Kirkland et al., 2005). Oxalate production reduces extracellular pH and, consequently, acts to facilitate degradation of components of the host cuticle, e.g., chitin, elastin and collagen (Bidochka and Khachatourians, 1991). *M. anisopliae* mutants unable to acidify the culture medium (i.e., that produced less metabolic acids) also show a decreased protease activity (St Leger et al., 1999). In *B. bassiana*, Luo et al. (2015) demonstrated that the *ΔBbmsn2* strain had little to no radial growth at pH 4.1 or 4.7 (although conidiation still occurred), and its growth was reduced in comparison to the wild-type strain. In addition, oosporein production is impaired in the Msn2 mutant (Luo et al., 2015), however its contribution to infection may be limited (Fan et al., 2017). A critical difference with respect to Msn2 function between insects and ticks may be that in insects Msn2 appears to contribute to both topical and intra-hemocoel infection (Luo et al., 2015), whereas our data indicate that for ticks, Msn2 is essentially only required for full virulence *via* the topical route of infection. While topical infection is the “natural” route of infection, these data imply important downstream immune system differences in dealing with invading (fungi) microbes between ticks and insects.

Effects on reproduction can be critical to the biological control potential of insect pathogenic fungi. Reduced fecundity and lack of resistance development have been reported in using *Cordyceps fumosorosea* (formerly, *Isaria fumosorosea*) against *Bemisia tabaci* (whitefly) (Gao et al., 2017), as well as in *B. bassiana* (Bernardo et al., 2018) or *M. anisopliae* (Bittencourt et al., 1994; Muniz et al., 2020) infecting *R. microplus* engorged females. After penetrating the cuticle of *R. microplus*, *M. anisopliae* reaches the hemocoel and can be found colonizing the hemolymph (Bittencourt et al., 1995) and internal organs, including the ovary tissues (Paulo et al., 2018) thus contributing to the reduced fecundity of engorged females seen after infection. *B. bassiana* infection towards *Argas persicus* (Acari: Argasidae) also directly affects the female reproductive system and causes damages to the ovaries, inhibiting vitellogenesis (Marzouk et al., 2020). Our data show that *B. bassiana* infection reduced fecundity of *R. microplus* engorged females by reducing ovipositing and larval hatching, with loss of Msn2 impairing these effects. For successful control, even if entomopathogenic fungi may not (quickly) kill engorged females, any reduction in fecundity can be crucial for controlling *R. microplus* infestations, because fully engorged females naturally drop off the host and lay thousands of eggs on the ground before dying and completing their life cycle.

In recent years, studies on the identification of genes and their functions in *B. bassiana* have revealed a wide range of mechanisms involved in the infection process (Butt et al., 2016); however, most of these studies have focused on insect larvae that are naturally more susceptible to infection by these fungi (Ortiz-Urquiza and Keyhani, 2016), and the extent to which these results can be extrapolated to other targets within Arthropoda remains to be determined. Our data indicate some shared contributions as well as potential differences. In addition, our results indicate that conidial germination, appressorial differentiation, and even fungal colonization on the host surface may be poor indicators of successful tick control (mortality), which requires host penetration (Ment et al., 2012).

## Conclusions

Our results indicate that the absence of Msn2 transcription factor reduced the virulence of *B. bassiana* s.l. against *R. microplus* demonstrated by the delayed fungal penetration and decreased protease production on the tick cuticle. Results on tick reproduction revealed potential effects beyond direct virulence that can impact biological control efforts.

## Data Availability Statement

The raw data supporting the conclusions of this article will be made available by the authors, without undue reservation.

## Ethics Statement

The animal study was reviewed and approved by Ethics Commission on Animal Use of Universidade Federal de Goiás (CEUA, protocol #057/16). The access to Brazilian genetic heritage was approved by the Genetic Heritage Management Council (CGen) of Brazil (protocol #A420934).

## Author Contributions

EM, NK and ÉF designed the experiments and wrote the manuscript. EM and CR-S performed the bioassays, histology of ticks and protease activity assays. EM and WA performed scanning electron microscopy and histology of ticks. NK inspired co-authors to investigate this subject and produced the mutant strains of *B. bassiana*. All authors contributed to the article and approved the submitted version.

## Funding

This study was supported by the Coordenação de Aperfeiçoamento de Pessoal de Nível Superior (CAPES) of Brazil for providing PhD scholarship for EM and CR-S. This research was supported by grants from the Conselho Nacional de Desenvolvimento Científico e Tecnológico (CNPq) of Brazil (431928/2016-9 to ÉF). CNPq also provided the grant 306319/2018-7 for ÉF. This research was also supported in part by US-NSF grant IOS-1557704 to NK. The publication costs were covered by grant from the Fundação de Amparo à Pesquisa do Estado de Goiás (FAPEG; CC 11233).

## Conflict of Interest

ÉF is Associate Editor for the section Fungi-Animal Interactions in Frontiers in Fungal Biology, and Guest Associate Editor for the section Invertebrate Physiology, in the research topic: Entomopathogenic Fungi for the Control of Arthropods - Frontiers in Physiology.

The remaining authors declare that the research was conducted in the absence of any commercial or financial relationships that could be construed as a potential conflict of interest.
